# Chinese College Students’ Attitudes towards Animal Welfare

**DOI:** 10.3390/ani12020156

**Published:** 2022-01-10

**Authors:** Sara Platto, Agathe Serres, Ai Jingyi

**Affiliations:** 1 Department of Biotechnology, College of Life Sciences, Jianghan University, No. 8, Sanjiaohu Rd., Wuhan Economic & Technological Development Zone, Wuhan 430056, China; 2 Institute of Deep Sea Science and Engineering, Chinese Academy of Sciences, 28 Luhuitou Road, Jiyang District, Sanya 572000, China; agathe.serres11@gmail.com; 3 Department of Translations, College of Foreign Language, Jianghan University, No. 8, Sanjiaohu Rd., Wuhan Economic & Technological Development Zone, Wuhan 430056, China; abigail_ai@163.com

**Keywords:** Chinese college students, animal welfare, animal sentient, five freedoms, college majors

## Abstract

**Simple Summary:**

College students, specifically from veterinary, animal, agricultural, and life sciences majors represent the future professionals who will closely work with animal industry stakeholders. Therefore, it is important to understand their attitudes towards animals and their knowledge about animal welfare. A survey on Chinese college students was conducted across different majors and Chinese geographical regions to understand their attitude towards the animal Sentient and the Five Freedoms models for pets, laboratory, farm, and wild animals. Most of the respondents exhibited a good attitude towards both the animal Sentient and the Five Freedoms models, with best scoring for pet animals followed by wild animals. Respondents showed less concerns towards farm and laboratory animals. A previous animal welfare education, the ownership of animals, and the participation in laboratory work involving animals positively influenced the attitude of the respondents towards animal welfare. These experiences might have helped students to develop concerns towards the animals’ treatment. When compared to previous studies, our results suggest that Chinese college students improved their attitudes towards animals in recent years.

**Abstract:**

Understanding the attitude of stakeholders towards animals is critical for the development and improvement of animal welfare in a country. College students from veterinary, animal, and life sciences majors represent future key stakeholders that will interact with professionals from animal industries. Therefore, it is critical to understand these college students’ attitudes towards animals and their knowledge about animal welfare. The present survey aimed to investigate Chinese college students’ concerns towards different animal classes (i.e., pets, farm, laboratory, and wild animals) through the animal Sentient and Five Freedoms models. Chinese college students from different majors (i.e., related to animal sciences or not) scored very well in their attitude towards both the animal Sentient and Five Freedoms models, with differences depending on the animal class considered. Pets (dogs and cats) had better consideration for both animal Sentient and Five Freedoms models, followed by wild animals, while farm and laboratory animals were less considered. Veterinary science major students showed the strongest differences in attitudes depending on the animal classes considered compared to other majors. Furthermore, respondents showed better attitude scoring if they currently owned or had owned animals, had participated in animal welfare courses, or in laboratory work that involved animals. When compared to previous studies, our results suggest a general improvement of Chinese college students’ attitudes towards animals.

## 1. Introduction

Animal welfare has attracted increased media attention in recent years, with society becoming more aware about the link between animals’ well-being and human health and the impact of the livestock industry on the environment [1]. Approximately fifty years ago, a science-based model was developed in order to capture the key aspects of animal welfare, namely the Five Freedoms model [2]. The Five Freedoms model was first formulated to describe the broader dimensions of animal welfare by incorporating subjective experiences, health status, and behavior [3,4,5]. On the other hand, the animal Sentient model refers to the notion that animals experience an array of emotions, from pain and suffering to pleasure and joy [6]. EU legally recognized animals as sentient beings since the enactment of the Treaty of Lisbon in 2007 [7], and till now, 32 countries have formally recognized non-human animals as sentient beings in their legislation [8]. In general, animal welfare is independent of the country where the animals are raised, but approaches to animal welfare may differ among countries also in relation to how different animal species are perceived [9]. The consideration of the physiological and behavioral needs of animals and the price a country is willing to pay to improve animal welfare can influence education, guidelines, and legislation developed to change the way animals are raised within a country [9,10]. 

China is one of the fastest growing economies in the world, and, like many other developing countries, it is strongly affected by the level of national economic development [11]. China is one of the largest producers of animal products, which was made possible by a wholesale adoption of “Western intensive modern” farming techniques such as gestation farrowing crates and battery cages, which have been banned in Europe since 2013 [12]. The use of these intensive farming practices, which have already been proven to threaten the welfare of the animals, is associated with China’s history on disease control measures; the use of substances in animal husbandry and food processing has lead Western countries to ban the import of certain Chinese animal products with an impact on the national economy [13,14,15]. In addition, there is a fundamental lack of understanding of the importance of animal welfare among the majority of Chinese animal industry stakeholders, leading to very little legislation and few interventions to address welfare issues [16,17]. This situation may be linked to the fact that the science of animal welfare originated in the Western countries, and this concept was only introduced in China mainland in the early 1990s, making it a very young discipline in this country [11,18]. For the last 20 years, China has tried to address animal welfare issues through some low level legislation regarding the aspects of farm animal rearing, transport, and slaughter, which are mainly based on food safety concerns [19]. The development and improvement of animal welfare measures and legislation in China require to first understand and improve its key stakeholders’ attitude towards animals.

During the last decade, in order to develop targeted actions to improve animal welfare, more attention has been paid to understand the attitude towards animals of stockpeople, veterinarians, researchers, university students, and consumers worldwide [13,17,20,21,22,23,24,25,26,27,28,29,30,31,32,33,34,35]. Different psychosocial factors have been identified as influencing people’s attitude towards animals [36]. These include animal traits (similarity to humans, cuteness, and vulnerability), individual human attributes (gender, age, educational level, early environment, experience with animals), and cultural factors (history, religious beliefs) [32,35,37]. 

Different surveys to assess the attitudes towards animal welfare of different animal industry stakeholders have been carried out in China in the past [17,19,38,39,40,41,42,43,44,45,46,47], including some on college students [13,48,49,50]. College students—particularly those from veterinary, animal, agricultural, and life sciences majors—represent the future generation of professionals who will work closely with animal industry stakeholders who will be influenced on how animals will be raised and treated. Therefore, it is critical to understand these students’ attitude towards animals and their knowledge about animal welfare in order to developed targeted educational programs depending of the areas of animals studies that need to be improved [51]. The first survey on Chinese college students carried out between 2002–2003 [49] revealed that a high percentage of respondents (90%) were concerned about the treatment of animals and their suffering, while a small percentage of students (15%) still supported the use and consumption of wildlife. It is important to keep in mind that this survey was conducted during the SARS epidemic in China, which might have caused a change of people’s perception towards animals, especially wildlife [49]. Moreover, Chinese college students from foreign language majors showed concerns with the treatment of animals in the country, in particular with animal experimentation, and they did not agree that humans could use the animals as they saw fit [48]. Even though these studies were carried out in affluent cities such as Beijing, Shanghai, and Guangzhou, where the residents’ high living standards may have biased the results, the findings of these surveys have still shown that Chinese society was changing its approach towards animal welfare [48]. Another survey that compared the attitudes towards animals of students from 13 Eurasian countries [13], showed that Chinese respondents had the lowest scores for the consideration towards animal welfare issues, even though a slight improvement of their perception towards animals was observed compared to past studies [45,46,48]. The study reported that almost half of the respondents from China never heard of the term “*animal welfare* [13]”. This could be explained by the fact that even Chinese legislation such as the Animal Husbandry Law (2005) did not include the term “*animal welfare*”, which underlines the fact that many legislators were of the opinion that animal welfare could not become a topic codified in the law [52]. Nevertheless, in the study of Phillips et al. [13], Chinese respondents still showed high concerns about wildlife protection, which might have been influenced by the cultural background and the place of origin of the students or an indication of the increased information regarding zoonosis transmission from wildlife to humans [13,53]. The recent COVID-19 pandemic has also affected the approach of Chinese society towards the human–animal relationship; despite the fact that the origin of the Sars-Cov-2 has not been yet determined, the epidemic has caused a dramatic change of attitude of the Chinese public in particular towards wildlife, including the government, who issued a new Wildlife Animal Protection legislation at the end of 2020. 

The results from such surveys are critical for shedding light on the possible obstacles that hindered the rise of animal welfare in China. For example, there are several factors that have been identified as possible obstacles to the improvement of animal welfare in China including (1) the Chinese government’s priority for economic development which might trump the animal welfare and protection actions; (2) the pressure of the increased meat consumption in the country, which might make it very difficult to reduce intensive farming practices; (3) the lack of well-defined animal welfare legislation in the country, which makes it difficult to impose national mandatory standards for animal practices [48]. Therefore, the assessment of the attitudes towards animals of Chinese animals’ industry stakeholders, from farmers to consumers, is critical to addressing specific needs and challenges in order to set up targeted actions for the improvement of animal welfare in the country. The current study assessed the attitude of Chinese college students from different majors towards the animal Sentient and Five Freedoms models for pet, farm, laboratory, and wild animals’ classes. 

## 2. Materials and Methods

### 2.1. Attitudes Scale Preparation

The scale to assess the Chinese college students’ attitudes towards animal welfare was designed following Mazas et al.’s [29] protocol for animal welfare attitude scale development. In the first step of the scale’s development, five components were defined: C1 farm animals, C2 pet animals (dogs and cats), C3 laboratory animals (monkey, mice, and dogs), C4 wild animals, and C5 animal Sentient/Five Freedoms models (for each of the animal classes considered). The component C1 included statements related to ways of treatment of farm animals and the different practices performed on them (ex: beak trimming, dehorning, hot iron branding). The C2 component included statements related to ways of treatment of dogs and cats and their potential suffering due to owners’ attitudes towards them (ex: abandonment, leaving the pet without water or food for few days). The C3 component included statements related to the treatment of animals used in research and their potential suffering due to experimentation. The C4 component included statements related to the treatment and management of wild animals (e.g., it is acceptable to kill wildlife because they transmit zoonosis). The C5 component included statements related to animal Sentient and Five Freedoms models for each animal class listed above. 

It is recommended to start with three or four times the number of items that will be included in the final scale [54]. Therefore, scale initially included 148 items. An item consisted in a statement and its associated Likert scale with six answers: (1) strongly agree, (2) agree, (3) neither agree nor disagree, (4) disagree, (5) strongly disagree, (6) I do not know. The scale underwent a two-way translation (English-Chinese Simplified; Chinese Simplified-English) to assess the validity of the translated items, and changes were made where discrepancies were evident. The translation was performed by two qualified translators familiar with the current study. 

The first step of the validation of the scale started with a panel of three experts who had to judge the validity of each item. The experts were selected within university professors from the disciplines of Chinese language, psychology, and animal welfare (veterinary medicine). During the second step of the validation of the scale, a small group of college students (*n* = 50) from different majors was asked to carefully examine each statement and to indicate which item was ambiguous and which one could not be rated. Following the students’ comments, some statements were modified while other kept the original wording. In the final step of the validation, the scale was submitted to a sample of 200 college students of different majors (animal and veterinary sciences, humanities, medicine, and hard sciences), and 107 completed questionnaires were returned. The purpose of this step was to detect the most problematic items, the difficulties in understanding the instruction, the errors in the format, and other possible mistakes [55]. 

An initial analysis of the items was carried out using SPSS 20 (IBM SPSS Statistics for Windows, Armonk, NY, USA). An item-total correlation and Cronbach’s Alpha coefficient analysis were calculated to eliminate problematic items and assess the reliability of the scale. Only items with a correlation between 0.3–0.6 were selected [56]. This first analysis resulted in the elimination of all the items included in the components C1, C2, C3, and C4, leaving only the items from the component C5 represented by the animal Sentient and Five Freedoms models. Following the Cormbach Alpha analysis, few items from the Five Freedoms and sentient models were excluded: (1) the item “*Animals are aware of their relationships with other animals and humans*” for the farm and wild animals’ classes, and the item “*Animals can learn from experience*” for the dog class in the animal Sentient model; (2) the item “*Animals should be free from hunger most of the time*” for the pets and wild animals classes in the Five Freedoms model. The obtained Chinese College Students Animal Welfare Attitude Scale (CCSAWAS) included 82 items (11 items for the animal Sentiente model for five animal classes, and 8 items for the Five Freedoms model for four animal classes) and had a Cronbach’s Alpha value of 0.98, which is considered a very good rating [57] (Table 1). The animal Sentient model included five animal classes: dog, cat, farm, laboratory, and wild animals, whereas the dog and cat classes were merged in a “pet” class for the Five Freedoms model. Initially, the sentient models items were included in each of the C1-C4 components that represented each of the animal classes considered in the study. In the C2 component, each item was listed separately for dog and for cat, as well for the Sentient model’s items. This latter format of the Sentient model was kept for the final version of the scale.

### 2.2. Structure of the Questionnaire

**Figure 1:**
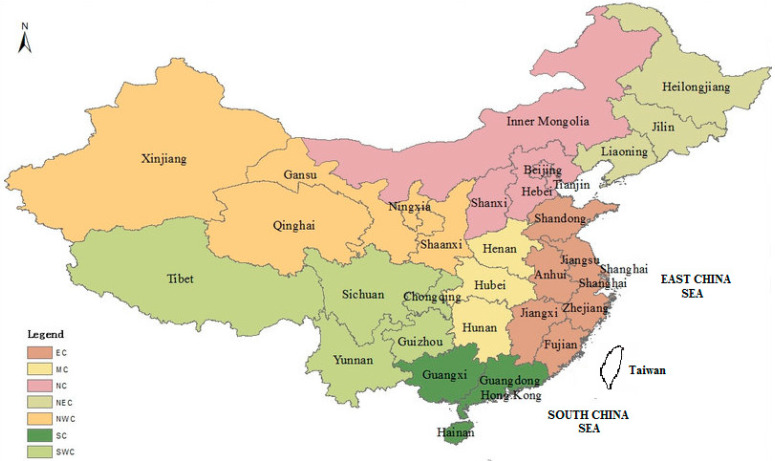
The map lists only the Chinese provinces included in the survey and grouped in geographical areas: East China: EC; South China: SC; Middle China: MC; North China: NC; Northwest China: NWC; Southwest China: SWC; North EastChina: NEC.

The first section of the questionnaire focused on demographic details such as the gender, age, year of study, major, family size (small: 1–3 people; medium: 4–5; large: 6–7), Chinese region of origin, and annual income (low: <24,000 RMB/year; medium: 24,000–60,000 RMB/year; upper medium: 60,000–120,000 RMB/year; high: >120,000 RMB/year). The mainland surveyed Chinese provinces were grouped by geographical location: East China (EC: Shandong; Jiangsu; Anhui; Zhejiang; Fujian; Shanghai; Jiangxi), South China (SC: Guandong; Guangxi; Hainan), Middle China (MC: Hubei; Hunan; Henan), North China (NC: Beijing; Tianjin; Hebei; Shanxi; Inner Mongolia), Northwest China (NWC: Ningxia; Xinjiang; Qinghai; Shaanxi; Gansu), South West China (SWC: Sichuan; Yunnan; Guizhou; Tibet; Chongqing), and North East China (NEC: Liaoning; Jilin; Heilongjiang) (Figure 1). Subsequently, the respondents were asked if they owned animals before the age of 18, if they currently owned animals, if they attended animal welfare classes, if they participated in laboratory work involving animals, and what their intended job was after graduation (Table 2). The rest of the questionnaire was structured with the list of the 82 items concerning the animal Sentient and Five Freedoms models for each of the animal classes considered for the study (pets, farm, laboratory, and wild animals). The items order was randomized throughout the questionnaire to avoid bias.

### 2.3. Survey Method

The questionnaire and the survey were approved by the Research Department of Jianghan University (Wuhan, China). The questionnaire was uploaded to the online platform Wèn Juàn Xīng (Changsha Ranxing Information Technology Co., Ltd, Changsha, China), which made it possible to obtain a weblink and QR code that was sent to students’ office of different universities in China through a national online platform. The questionnaire was uploaded on the platform where students throughout China take their exams to apply for undergraduate and graduate studies every year. The students who answered the questionnaire were given additional points for their final exam score. The total questionnaire required no more than 10 min of time to be completed. The respondents were able to access the questionnaire by using their phones, computers or tablets. The uploaded questionnaire had an initial part that explained the purpose of the survey and anonymity of the responses. The link remained open from January until September 2020, allowing the students to complete the survey at their convenience.

### 2.4. Statistical Analysis 

Statistical analyses were performed with the program R, version 4.0.5 [58]. “I don’t know” answers were first transformed into NAs in order to not impact scores. Other responses were transformed into numbers (strongly agree = 1, agree = 2, not agree nor disagree = 3, disagree = 4, and strongly disagree = 5). Therefore, lower scores (1, 2) indicated positive attitudes towards the variables considered; higher scores (4, 5) indicated negative attitudes towards the variables considered; a middle score (3) indicated neither positive nor negative attitudes towards the variable considered. The score 6 (I do not know) indicated uncertainty towards the variable considered. 

The 82 items were grouped following the two major models, sentient and Five Freedoms. Each item was repeated for each animal category (sentient model: dogs, cats, farm animals, lab animals, wild animals; Five Freedoms model: pets, farm animals, laboratory animals, wild animals). Answers were averaged for these two concepts and two linear mixed effect models were run with the “lmer()” function from the “lme4” package [59]. In addition, the rate of “I don’t know” answers was calculated to obtain an “uncertainty rate” for each student. The influence of the students’ characteristics on this rate was analyzed through a logistic regression for quasi-binomial data using the “glm()” function from the “stats” package.

The three models included the average attitude score (towards animals’ sentient and towards the five freedom models), and the uncertainty rate as the response variable. The students’ gender (female or male), age (below 20, between 20–25, or over 25 years old), region of origin (East China, South China, Middle China, North China, Northwest China, Southwest China, or Northeast China), income (low, medium, upper medium, or high), family size (small, medium, or large), majors (life science, veterinary medicine, humanities/art/education, business/finance/law, human health—medicine/psychology—nursing school, engineering/IT/Science—math and physics), year of studies (Freshman, Junior, Senior, Graduate school, or Sophomore), intended career (animal-related, medicine/research-related, education-related, or other) were added as predictors as well as their declarations about owning animals before 18 (yes or no), currently own animals (yes or no), attending welfare lectures in the past (yes or no), and experienced laboratory experiments on animals (yes or no), and the animal category mentioned in the question (pet, farm, laboratory, and wild animal). The ID of the students was also added as a random factor to account for repeated measurements. The interaction between major and animal category and between major and year of studies was included in linear mixed effect models.

For each of the linear mixed effect models, a detailed model diagnosis was conducted to check the normality of residuals and the homogeneity of variances. After this diagnosis, the attitude score averages were log-transformed to correct for normality of residuals’ issues. Multicollinearities were then checked using a generalized variance inflection factor (GVIF(1/(2× Df) [60], using the “corvif” function from the AED package [61], with no major issues (no GVIF(1/(2× Df)) > 2). A stepwise model selection was achieved and the model with the lowest Akaike Information Criterion (AIC) was selected. Wald chi-squared tests were used to obtain *p*-values. Pairwise tests were conducted by running the same models with appropriate sub-settings and applying a Bonferroni correction. Pairwise comparisons that were not significant after application of the Bonferroni correction will not be mentioned. Means and confidence intervals presented in figures or tables have been extracted from models and back-transformed from a log (for linear mixed effect models) or logit (for logistic regressions) scale.

## 3. Results

A total of 5795 completed questionnaires were collected. 77.1% of the students supported the idea that animals have sentient characteristics, while 2.7% did not agree at all. 76.8% of the students agreed that animals should be guaranteed basic animal welfare standards, while only 2.7% did not support animal welfare at all. Chinese college students gave more uncertainty answers (“I don’t know”) for the animal Sentient model (69.2%) than for the Five Freedoms model (30.8%). They attributed the highest sentient characteristics to dogs (82%), followed by cats (79%), wildlife (77%), farm animals (74%), and laboratory animals (73%). Similar results were found for the Five Freedoms model, with more concerns towards pets (79%), and wild animals (79%), followed by farm animals (78%), and laboratory animals (71%). In general, students showed the lowest uncertainty rate for wild animals (21.1%), followed by pets (22.2%), and farm animals (25.5%), with the laboratory animals getting the higher percentage (31.3%) for the Five Freedoms model. Similar results were found for the Sentient model, where dogs had the lowest percentage of uncertainty rate (13.3%), followed by wild animals (18%), cats (20.8%), farm animals (22.2%), and laboratory animals (25.9%). 

### 3.1. Attitude towards the Animal Sentient Model

**Figure 2:**
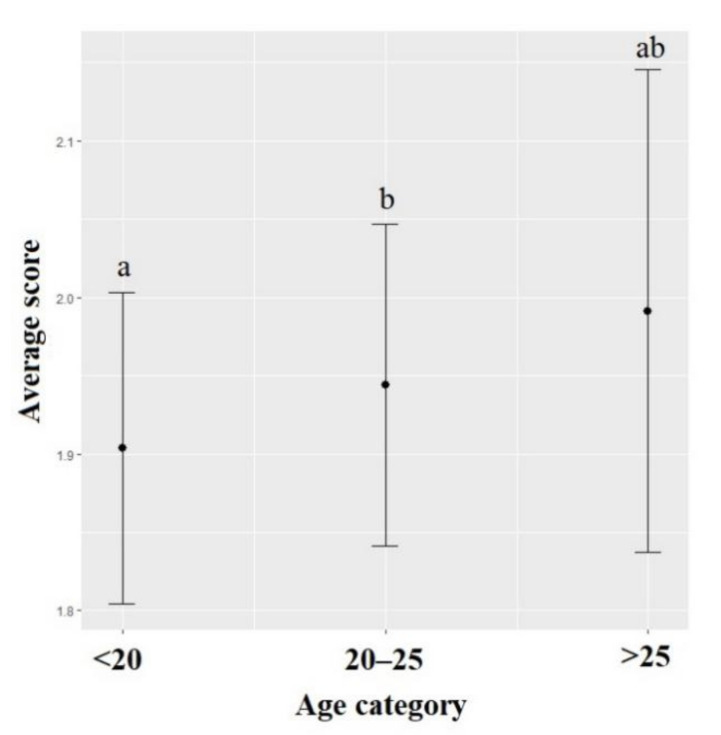
Means and 95% confidence intervals of scores towards the animal Sentient model depending on the students’ age. Means and 95% confidence intervals have been back-transformed from a log scale. Different letters represent significant differences (Wald chi-square with Bonferroni correction).

**Figure 3:**
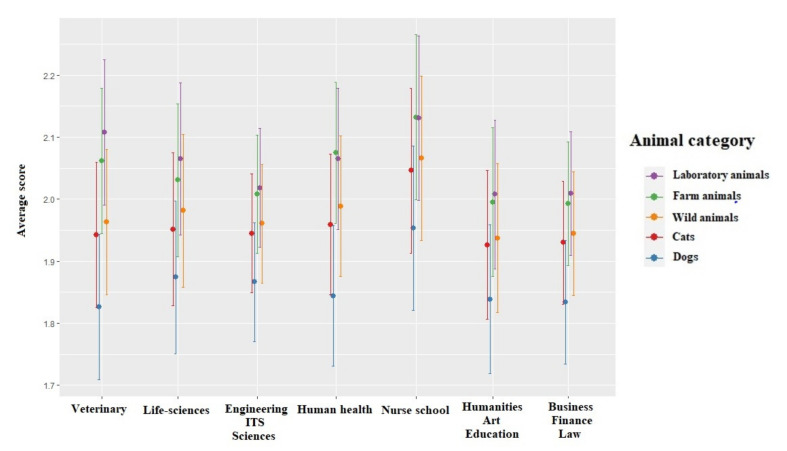
Means and 95% confidence intervals of scores towards the animal Sentient model depending on the students’ major and the animal class. Means and confidence intervals have been back-transformed from a log scale, but 95% confidence intervals are presented on the log scale.

The scores for the attitude towards animals’ sentient were significantly lower for females than for males (χ^2^ = 11.180, df = 1, *p* < 0.001). These scores were also significantly impacted by the students’ age (χ^2^ = 5.11, df = 2, *p* = 0.024): students that were younger than 20 years old had lower scores than students between 20–25 years old (χ^2^ = 3.850, df = 1, *p* = 0.049) (Figure 2). The region the students originated from significantly impacted the attitude toward animals’ sentient (χ^2^ = 21.343, df = 6, *p* = 0.002) with scores of students from Southwest China being significantly higher than those of students from Northwest China (χ^2^ = 4.057, df = 1, *p* = 0.001). The annual income (χ^2^ = 40.491, df = 3, *p* < 0.001) also significantly impacted this score with lower scores for students with a high income than for those with a medium (χ^2^ = 5.404, df = 1, *p* < 0.001) or low income (χ^2^ = 4.454, df = 1, *p* < 0.001). The scores for the attitude towards animals’ sentient were significantly lower for students who owned animals before 18 (χ^2^ = 106.440, df = 1, *p* < 0.001), who currently own animals (χ^2^ = 14.757, df = 1, *p* < 0.001), who assisted to welfare lectures (χ^2^ = 17.402, df = 1, *p* < 0.001) and who did laboratory experiments with animals (χ^2^ = 5.294, df = 1, *p* = 0.021) (Table 3) than students who did not. The attitude towards animals’ sentient did not significantly vary depending on the students’ year of studies (χ^2^ = 5.990, df = 4, *p* = 0.199), family size (χ^2^ = 3.038, df = 2, *p* = 0.218), or intended career (χ^2^ = 1.849, df = 3, *p* = 0.604). The interaction between the major and the animal category was significant (χ^2^ = 45.319, df = 6, *p* < 0.001), with similar patterns among animal categories for all students (laboratory and farm animals obtained the highest scores while dogs obtained the lowest), but students from different majors exhibited different degree of animal category bias, with scores being the most different among animal categories for veterinary students (Figure 3).

### 3.2. Attitude towards the Five Freedoms Model

**Figure 4:**
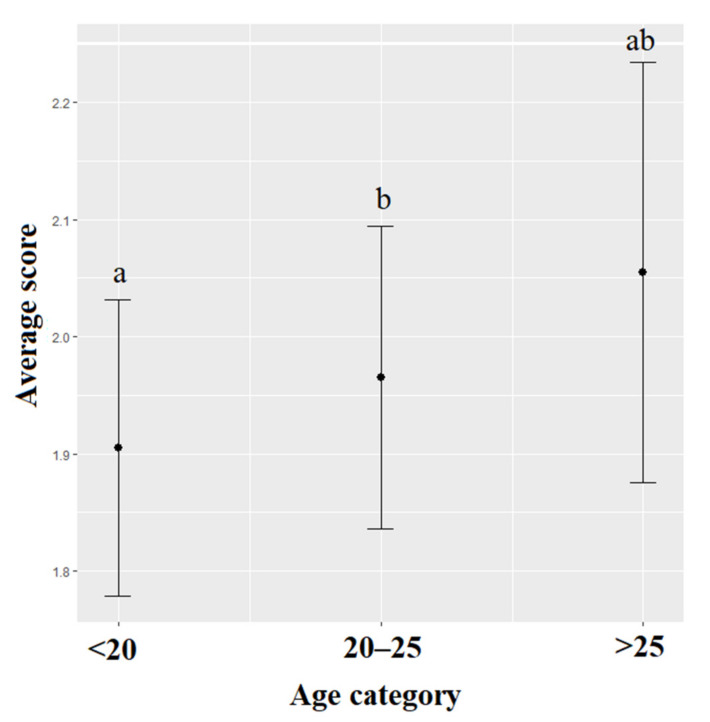
Means and 95% confidence intervals of scores towards the Five Freedoms model depending on the students’ age. Means and 95% confidence intervals have been back-transformed from a log scale. Different letters represent significant differences (Wald chi-square with Bonferroni correction).

**Figure 5:**
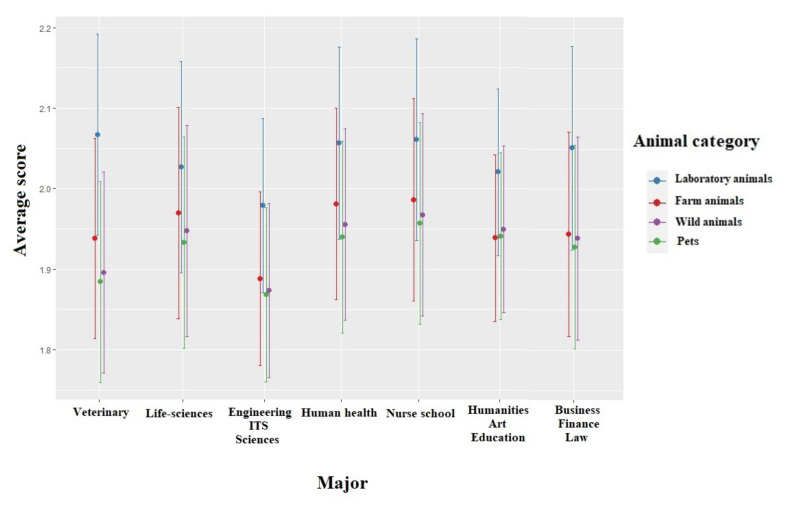
Means and 95% confidence intervals of scores towards the Five Freedoms model depending on the students’ major and the animal class. Means have been back-transformed from a log scale but 95% confidence intervals are presented on the log scale.

The scores for the attitude towards the five freedoms were significantly lower for females than for males (χ^2^ = 19.388, df = 1, *p* < 0.001). These scores were also significantly impacted by the students’ age (χ^2^ = 10.328, df = 2, *p* = 0.001): students that were younger than 20 years old had lower scores than students between 20–25 years old (χ^2^ = 8.038, df = 1, *p* = 0.005) (Figure 4). The region the students originated from significantly impacted the attitude toward animals’ sentient (χ^2^ = 26.186, df = 6, *p* = 0.002) with scores of students from Southwest China being significantly higher than those of students from Northwest China (χ^2^ = 4.057, df = 1, *p* = 0.001). The annual income (χ^2^ = 53.801, df = 3, *p* < 0.001) also significantly impacted this score with lower scores for students with a high income than for those with a medium (χ^2^ = 5.404, df = 1, *p* < 0.001) or low income (χ^2^ = 4.454, df = 1, *p* < 0.001). The scores for the attitude towards the five freedoms were significantly lower for students who owned animals 18 (χ^2^ = 105.675, df = 1, *p* < 0.001), who currently own animals (χ^2^ = 12.341, df = 1, *p* < 0.001), who assisted to welfare lectures (χ^2^ = 12.929, df = 1, *p* < 0.001) and who did laboratory experiments with animals (χ^2^= 5.223, df = 1, *p* = 0.022) than students who did not (Table 4). The attitude towards five freedoms did not significantly vary depending on the students’ family size (χ^2^ = 4.794, df = 2, *p* = 0.091) or intended career (χ^2^ = 3.411, df = 3, *p* = 0.332). The interaction between the major and the animal category was significant (χ^2^ = 27.232, df = 6, *p* < 0.001), with similar patterns among animal categories for all students (laboratory and farm animals obtained the highest scores while pets obtained the lowest) but students from different majors exhibited different degree of animal category bias, with scores being the most different among animal categories for veterinary students (Figure 5). 

### 3.3. Uncertainty Rate 

The uncertainty rate was not significantly impacted by the students’ gender (χ^2^ = 10.063, df = 1, *p* = 0.435). This rate was significantly impacted by the students’ age (χ^2^ = 6.224, df = 2, *p* = 0.044), but pairwise comparisons were not significant. The region the students originated from did not significantly impact the uncertainty rate (χ^2^ = 9.037, df = 6, *p* = 0.172). The annual income (χ^2^ = 9.581, df = 3, *p* = 0.022) and family size (χ^2^ = 9.081, df = 2, *p* = 0.007) significantly impacted this rate, but pairwise comparisons were not significant. The uncertainty rate was not significantly impacted by the students’ major (χ^2^ = 0.103, df = 1, *p* = 0.748) or intended career (χ^2^ = 2.693, df = 3, *p* = 0.441). The uncertainty rate was significantly lower for students who owned animals before 18 (χ^2^ = 21.043, df = 1, *p* < 0.001), who attended welfare lectures (χ^2^ = 8.207, df = 1, *p* = 0.004) and who did laboratory experiments with animals (χ^2^ = 7.268, df = 1, *p* = 0.007) than for students who did not. The uncertainty rate was not significantly influenced by the current ownership of animals (χ^2^ = 0.157, df = 1, *p* = 0.691) (Table 5).

## 4. Discussion

In line with previous surveys [13,48,49,50], the current study showed that the majority of Chinese college students’ supported the idea that the animals of the classes considered (pets, farm, laboratory, and wild animals) have sentient characteristics, and that they should be guaranteed basic animal welfare standards, while only a small percentage (2.7%) did not agree with both models considered. Higher uncertainty rates were found for items related to the animal Sentient model (69.2%) compared to the Five Freedoms model (30.8%). These results might suggest that the concept of animal welfare and needs may be better understood than the sentient aspects of animals. Therefore, the development of courses within Chinese universities that increase the awareness of animals’ capacity to experience emotions or possess certain traits present in humans is needed. Considering animals as sentient beings would probably result in more consideration for their welfare. 

Among students, females showed better attitudes towards animals than males. Significant gender differences exist in animal welfare attitudes in the literature [13,19,28,62,63,64,65,66,67]. In general, females represent the primary family caretakers, which makes them more prone to develop positive attitudes that can go beyond the family care and be extended to animals [68]. The psychological literature demonstrated that females tend to be more empathetic than males towards both humans and other animals, showing greater concern for their welfare and suffering, while males have the tendency to assume a more “dominionistic attitude” towards it [35,69,70,71,72]. In addition, since females are more engaged with animals than males are, they are more keen to believe that animals experience emotions, while males tend to be more skeptic about this concept [72]. The unbalanced male-to-female ratio (1:4) in the present study may also have impacted the results. 

In line with previous studies on college students from Western countries [32,33,73,74], younger Chinese respondents exhibited better attitudes towards animals than older ones. These findings could be attributed to the fact that younger students are not yet affected by the hardening of the study that is observed in certain scientific majors (veterinary, animal, agricultural, life sciences, and medicine) in advanced years, which often involves practices on animals [75,76]. In contrast, Levine et al. [26] found that younger students scored worse in attitudes towards animals than older ones. The authors explained that younger students might not have yet been involved in animal welfare classes, which could influence their knowledge and attitudes towards animals. The Chinese government introduced animal welfare classes (non-mandatory) in high schools in 2018, which could explain why, in the current study, younger respondents scored better than older ones.

It is believed that the living place, including the environment and type of work practices can shape the attitudes of the people [68,77], including their attitudes towards animals. The present study showed that Chinese college students from the Northwest region showed better attitudes towards animal Sentient and Five Freedoms models than those coming from the Southwest region. The provinces from Northwest China are mostly characterized by arid (desert) and semi-arid environments where farmers mainly breed small ruminants and camels, with farming activities being mostly represented by pastoral systems and small back-yard farms. Small-scale farms and pastoral systems could create proximity with animals, predisposing habitants to exhibit better attitudes towards animals [78]. In addition, this region has also undergone a series of sustainable developmental projects in the last thirty years in order to alleviate poverty by providing incomes from wildlife management and environmental protection [79,80]. On the other hand, the provinces from Southwest China are characterized by human-animal conflicts, and they also include major wildlife trafficking routes from Southeast Asia [81,82]. Provinces from Southwest China, in particular Yunnan, contain China’s largest tropical forest, and are characterized by an imbalance between nature conservation and economic development that have led to increased conflicts between humans and Asian elephants (*Elephas maximus*) for instance [81]. Poaching activities are still ongoing in these areas, and being habituated to such practices that may be linked to bad attitudes towards animals [80,81,82]. 

Family income has been found to be a predictor of attitude towards animals with opposite results depending on the study. For example in a study, respondents from families with lower income scored better in their attitudes towards animals [68], while a different survey showed that higher education and higher income were predictors of better attitude towards animals [83]. In our study, Chinese college students coming from higher income families and lower number of family members exhibited better attitudes towards animals both for the animal Sentient and the Five Freedoms models. The respondents of the current survey are living in a country that is much richer and with stronger societal changes compared to the reality where their parents grew up [48], and this situation may generate a feeling of well-being which might compel people to extend the same condition to animals [84,85]. 

Chinese college students who owned an animal during their childhood or at the time of the survey showed better attitudes towards animals and exhibited lower uncertainty rates than those who did not own animals at all. In general, affectionate childhood interactions with animals are associated with more humane attitudes towards them [35,86,87,88], while more exploitative or consumptive interactions early in life or during adulthood tend to result in more utilitarian perspectives [88,89]. This result emphasizes the importance of having young generations interacting positively with animals in order to develop a sense of respect and protection towards them. Unfortunately, in this study it was not possible to assess the type of animal the students owned currently or during their childhood, which would have given more information on how this factor would affect their attitudes towards animals.

The students’ attitude differed depending on the animal class considered. The striking outcome was that students from all majors had better consideration towards pets, with dogs being the class considered as the most sentient. This result is consistent with the findings of the study of Phillips et al. [13], where Chinese respondents gave dogs a high sentient capacity (even higher than human infants). China has the world’s largest dog and cat population with an estimated 180 million companion animals and approximately 2% of Chinese households owing dogs, and 6% owing cats [90,91]. The amount of money spent on companion animals has also doubled over the past decade [92,93]. In addition, dog eating is rejected by the majority of the Chinese young generations, many of whom are very active in animal protection organizations [94]. Therefore, young Chinese generations should be encouraged to pursue further education and activities that support the welfare of dogs and cats in China in order to favor more improvements of the field, such as the establishment of a pet animals’ protection legislation that is still missing in the country. 

Furthermore, in line with previous surveys conducted on Chinese students [13,43,48,49], the respondents of the current study showed positive attitudes towards wild animals, which was also reflected by a lower uncertainty rate. Shuxian at al. [49] have pointed out that the epidemic of SARS in China in 2003 might have influenced a positive change in the societal attitudes towards wildlife eating and animal treatment in general. An increase of information regarding zoonosis (i.e., diseases transmitted from animal to human) could explain the raise of interest towards wildlife welfare [17]. A similar situation might have also happened in the current survey, which was carried out when China experienced the first outbreak of COVID-19, which has been subjected to a huge media exposure, including discussions on the illegal wildlife trade that is still considered the main trigger of the pandemic. Therefore, wildlife protection is becoming a very complex and challenging public policy issue in China, and an increase of concerns towards the welfare of wild animals is observed in young generations [45,46,47,95]. 

Regarding farm animals, Chinese people have been found to consider the welfare of this animal class as an attribute of the food safety and quality concepts, with more importance given to these than to other attribute specifically related to animals [96,97,98]. Animals regarded with positive emotions (dogs and cats), or that display human-like behaviors or represent beauty symbols tend to be exempt from harmful exploitation [32,99,100], while “utility animals” such as farm animals do not seem to evoke high empathetic responses with subsequently low considerations towards them [35,101,102]. Therefore, more education regarding the conditions of farm and laboratory animals should be further developed. 

Finally, Chinese college students showed the least concern for farm and laboratory animals. A previous study showed the Chinese college students had a certain concern for the use of animals in experimentation [50]. Students agreed that the use of laboratory animals for testing of cosmetics and household products was unnecessary and should be stopped. In addition, they did not agree with the statement that humans had the right to dispose of animals as they saw fit [50]. Nonetheless, the level of concern of the students towards laboratory animals in the current study was still moderate. It is interesting to notice that, despite this lower level of concerns towards laboratory animals by Chinese college students, the *Guidance on **K**ind treatment of **L**aboratory **A**nimals* legislation issued in 2006 in China, is the only one that recognizes the Five Freedoms and the avoidance of unnecessary suffering to animals [103]. In addition, several Chinese airlines (AirChina, China Southern, China Eastern) announced in March 2012 the suspension of the transport of animals used in research, as the public began to oppose to the use of animals in experimentation under the widespread publicity of PETA [11]. This might also explain why in the current study, the participation in laboratory work involving animals was associated with better attitudes towards animal Sentient and the Five Freedom models and lower uncertainty rates compared to students who did not performed lab work with animals. Students who participated in laboratory work with animals might have developed some concerns related to the animals’ treatment during experimentation. 

Chinese students from veterinary, human health, and nurse majors showed the highest differences in scoring depending on the animal class. Previous studies found that respondents from scientific majors such as veterinary, agricultural sciences, pharmacy, and medical schools were more likely to accept the use of animals for experimentation [96]. A previous study found that veterinary students showed lower considerations towards farm animals, believing that this animal class did not experience pain in the same way other animals, such as pets, do [26]. They were also found to be more likely to believe that dogs and cats had more cognitive abilities than farm animals, and to consider various procedures to be more humane for farm animals than for pets [26,51]. These outcomes should seriously be taken into consideration to develop specific educational programs for Chinese students of the majors that are related to animals in order to reduce the animals class bias. 

Chinese college students who participated in animal welfare lectures showed better attitude towards animals and had lower uncertainty rate than those who did not. Participation in animal welfare courses appears to have a number of positive effects on students’ knowledge and attitudes towards animals. In a previous study, students enrolled in the animal welfare courses were able to identify more of the factors that have an impact on animal welfare (biological functioning, ability to exist in natural state, feelings), demonstrating that the courses were effective in helping students to better appreciate the broad range of criteria that should be used in assessing animal welfare [62]. Such animal welfare lectures may benefit to become mandatory.

In previous studies, early year students—in particular from scientific majors such as agriculture, veterinary medicine, and medical school—showed better attitudes and empathy towards animals compared to the students of later years who were more accepting of certain practice towards animals [76,104,105]. The lack of differences in attitudes depending on the years of study in the current survey might have been caused by the fact that almost three quarter of the respondents were freshmen and sophomore students. In addition, another study found that the most influential factor on the students’ attitude towards animals was not the year of study but the age [82], which may have been the case here. Moreover, students’ career choices also did not seem to affect the attitudes of the respondents towards animals, but respondents who intended to work with animals scored slightly better than students who chose other career types. This is in line with previous hypothesis suggesting that attitudes towards animals might develop independently of career aspiration or future career goals [26]. 

## 5. Conclusions

The current study represents the first comprehensive assessment of Chinese college students’ attitudes towards animal welfare across different majors and Chinese universities (596 Chinese academic institutions between colleges and universities), and all geographical regions of the country. In fact, past studies that performed similar surveys in China, considered only respondents from a small pool of universities [49], or they were limited to few or one major Chinese city [48,49,50]. Even though, the present survey does not represent the opinions and attitudes towards animals of all Chinese college students, considering the small number of respondents assessed (5975) compared to the overall national population of college students in the country (32.9 millions) [106], the results still provide a good view of the position that Chinese young generations have towards animal welfare.

Chinese college students showed good scoring for both animal Sentient and Five Freedoms models for all animal classes considered (pets, farm, laboratory, and wild animals). Female and younger students scored better than males and older students. Regardless of their majors, students showed greatest concerns for the welfare of dogs, followed by cats, wild, farm and laboratory animals. The good scores and low uncertainty rates found for wild animals may reflect a possible change in attitudes of Chinese young generation towards this animals’ class. In addition, students who owned animals during their childhood and who attended animal welfare classes or laboratory work that involve animals showed better attitudes, and had lower uncertainty rate compared to those who did not. We suggest that implementing more welfare lectures for all students, with a focus on laboratory and farm animals, could be beneficial to the global attitude of Chinese people towards animals. Students from an animal-related major should be even more targeted to reduce the bias they have depending on the animal class that may impact their future work.

It is important to take into consideration that the surveyed Chinese students grew up with better material conditions, education opportunities, and more influence from Western media than older generations, and therefore they might have developed a stronger concern towards animals than older people. The younger Chinese generation is getting more involved in animal protection thanks to the presence of the Chinese Small Animal Protection Association, which is one of the few animal protection organizations recognized by the Chinese government and which has representative offices in every university campus in China. Chinese college students of all majors can volunteer in this organization, whose main work is educating the public on the small animal protection and husbandry (mainly dogs and cats) and rescue of stray animals. We suggest that the participation in such organization should be encouraged. The results of the current survey suggest that future Chinese generations may help improve the treatment of the animals in the country and participate in the development of a more solid legislation that supports the welfare of all animal classes. However, to ensure this improvement, animal welfare, including animal Sentient and the Five Freedoms should be focused during animal welfare lectures. These lectures should not be limited to discussions about pets, but include all animal classes and should encourage young Chinese to get involved in animal-related organizations.

## Figures and Tables

**Table 1:** List of items for Five Freedoms and animal Sentient models used in the scale.

Animal Sentient Model
Animals are emotionally influenced by the conditions of their mates
Animals are aware of their surroundings
Animals can experience positive or negative emotions in the same way that humans do
Animals are able to choose among different individuals of the same species, objects, and situations
Animals are aware of what is happening to them
Animals are able to have emotional bonds with humans
Animals are aware of their bodily sensation (pain, hunger, heat, cold)
Animal can develop emotional bonds with individuals of the same species
Animals are aware of their relationships with other animals and humans
Animal feel pain in the same way that humans do
Animals can learn from experience
**Five Freedoms Model**
Animals should be free from unnecessary fear and distress
Animals should be free to express the majority of their normal behaviors repertoire
Animals should be free to interact naturally with other animals as their nature dictates
Animals should be free from thirst most of the time
Animals should be free from unnecessary suffering (emotional and physical)
Animals should have enough space to lie down and move freely
Animals should be free from injuries and disease (or prompt treatment should arise)
Animals should be free from hunger most of the time

**Table 2:** Demographics of respondents to the Chinese College Students Animal Welfare Attitude Scale questionnaires analyzed.

Demographic Variables		*n*. of Respondents	% of Survey Sample
**Gender**	Male	4158	71.8
	Female	1619	27.9
	NA *	17	0.3
**Age**	<20	2312	39.9
	20–25	3396	58.6
	>25	68	1.2
	NA *	19	0.3
**Year of study**	Freshman	2755	47.5
	Sophomore	2082	35.9
	Junior	681	11.8
	Senior	224	3.9
	Graduate	53	0.9
**Major**	Veterinary medicine	332	5.7
	Life-sciences	474	8.2
	Engineering/IT/Sciences	1298	22.4
	Human health	661	11.4
	Nurse school	1158	20
	Humanities/Art/Education	909	15.7
	Business/Finance/Law	963	16.6
**Region of origin**	East China	1666	28.8
	South China	230	4
	Middle China	622	10.7
	North China	577	9.9
	North-West China	1496	25.8
	South-West China	888	15.3
	North East China	115	2
	NA *	201	3.5
**Income**	Low	2409	41.6
	Medium	1458	25.2
	Upper medium	687	11.9
	High	333	5.7
	NA *	908	15.7
**Family Size**	Small	1557	26.9
	Medium	2306	39.8
	Large	1855	32
	NA *	77	1.3
**Intended Career**	Animal-related	378	6.5
	Medical/research	1572	27.1
	Education	418	7.2
	Other	2473	42.7
	NA *	954	16.5
**Owned animals before 18 years old**	No	2460	42.5
	Yes	3335	57.5
**Currently owning animals**	No	4531	78.2
	Yes	1264	21.8
**Attended animal welfare classes**	No	5192	89.6
	Yes	603	10.4
**Did laboratory work on animals**	No	4587	79.2
	Yes	1208	20.8

NA *: no data (the respondents did not answer that particular item of the questionnaire).

**Table 3:** Means and 95% confidence intervals (CIs) of scores towards animal Sentient model according to the students’ characteristics. Within each factor, categories that have different lower-case letters differ significantly (Wald chi-squared tests with Bonferroni correction). *n*= number of students.

Factor	Level	Fitted Mean	Lower CI	Upper CI	*n*
**Gender**	Female ^a^	1.86	1.82	1.89	4158
	Male ^b^	1.91	1.87	1.95	1619
**Region**	Northwest ^a^	1.87	1.75	1.99	1496
	South	1.85	1.72	1.99	230
	North	1.87	1.75	2	577
	East	1.9	1.79	2.02	1666
	Middle	1.92	1.79	2.04	622
	Northeast	1.94	1.79	2.09	115
	Southwest ^b^	1.97	1.85	2.09	888
**Income**	Low ^a^	1.97	1.86	2.09	2409
	Medium ^a^	1.95	1.84	2.07	1458
	Upper medium	1.89	1.77	2.01	687
	High ^b^	1.81	1.68	1.93	333
**Family size**	Small	1.78	1.68	1.89	1557
	Medium	1.84	1.78	1.9	2306
	Large	1.86	1.81	1.91	1855
**Major**	Veterinary medicine	1.86	1.78	1.94	332
	Life-sciences	1.88	1.66	2.11	474
	Engineering/ITS/Sciences	1.84	1.7	1.97	1298
	Human health	1.84	1.7	1.98	661
	Nurse school	1.86	1.69	2.03	1158
	Humanities/Art/Education	1.76	1.54	1.97	909
	Business/Finance/Law	1.74	1.64	1.84	963
**Intended career**	Animal-related	1.81	1.73	1.89	378
	Medical/research	1.84	1.78	1.9	1572
	Education	1.83	1.76	1.89	418
	Other	1.83	1.77	1.9	2473
**Owned animals before 18 years old**	No ^a^	1.91	1.85	1.98	2460
	Yes ^b^	1.74	1.68	1.8	3335
**Currently owning animals**	No ^a^	1.87	1.81	1.93	4531
	Yes ^b^	1.79	1.72	1.85	1264
**Attended animal welfare lectures**	No ^a^	1.87	1.81	1.93	5192
	Yes ^b^	1.78	1.71	1.85	603
**Did laboratory work on animals**	No ^a^	1.85	1.79	1.91	4587
	Yes ^b^	1.8	1.74	1.87	1208
**Animal category**	Laboratory animals	1.94	1.85	2.03	NA
	Farm animals	1.91	1.82	2	NA
	Wild animals	1.84	1.75	1.93	NA
	Cats	1.82	1.73	1.91	NA
	Dogs	1.72	1.63	1.81	NA

**Table 4:** Means and 95% confidence intervals (CIs) of scores towards the Five Freedoms model according to the students’ characteristics. Within each factor, categories that have different lower-case letters differ significantly (Wald chi-squared tests with Bonferroni correction). *n*= number of students.

Factor	Level	Fitted Mean	Lower CI	Upper CI	*n*
**Gender**	Female ^a^	1.62	1.43	1.81	4158
	Male ^b^	1.7	1.51	1.89	1619
**Region**	Northwest ^a^	1.61	1.42	1.8	1496
	South	1.62	1.42	1.83	230
	North	1.63	1.44	1.83	577
	East	1.68	1.49	1.87	1666
	Middle	1.68	1.48	1.87	622
	Northeast	1.68	1.46	1.9	115
	Southwest ^b^	1.73	1.54	1.92	888
**Income**	Low ^a^	1.76	1.51	1.89	2409
	Medium ^a^	1.7	1.45	1.83	1458
	Upper medium	1.64	1.57	1.95	687
	High ^b^	1.55	1.35	1.75	333
**Family size**	Small	1.61	1.4	1.81	1557
	Medium	1.68	1.49	1.87	2306
	Large	1.7	1.51	1.89	1855
**Major**	Veterinary medicine	1.65	1.45	1.85	332
	Life sciences	1.67	1.47	1.87	474
	Engineering/ITS/Sciences	1.60	1.41	1.8	1298
	Human health	1.68	1.49	1.88	661
	Nurse school	1.69	1.5	1.89	1158
	Humanities/Art/Education	1.66	1.47	1.86	909
	Business/Finance/Law	1.67	1.47	1.87	963
**Intended career**	Animal-related	1.62	1.43	1.82	378
	Medical/research	1.68	1.49	1.87	1572
	Education	1.65	1.46	1.85	418
	Other	1.68	1.48	1.87	2473
**Owned animals before 18 years old**	No ^a^	1.75	1.56	1.94	2460
	Yes ^b^	1.57	1.38	1.76	3335
**Currently owning animals**	No ^a^	1.7	1.51	1.89	4531
	Yes ^b^	1.62	1.43	1.81	1264
**Attended animal welfare lectures**	No ^a^	1.71	1.52	1.9	5192
	Yes ^b^	1.6	1.41	1.8	603
**Did laboratory work on animals**	No ^a^	1.68	1.49	1.87	4587
	Yes ^b^	1.64	1.44	1.83	1208
**Animal category**	Pets	1.62	1.43	1.81	NA
	Wild animals	1.63	1.44	1.82	NA
	Farm animals	1.65	1.46	1.84	NA
	Laboratory animals	1.75	1.56	1.94	NA

**Table 5:** Means of uncertainty rate and 95% confidence intervals (CIs) according to the students’ characteristics. Within each factor, categories that share the same letter do not differ significantly, categories that have no letter in common differ significantly or tend to differ (Wald chi-squared tests with Bonferroni correction). *n* = number of students.

Factor	Level	Fitted Mean	Lower IC	Upper IC	*n*
**Gender**	Female	0.010	0.006	0.017	4158
	Male	0.011	0.006	0.020	1619
**Age**	<20	0.006	0.004	0.011	2312
	20–25	0.008	0.005	0.014	3396
	>25	0.021	0.007	0.060	68
**Region**	North	0.006	0.003	0.015	577
	Middle	0.006	0.002	0.016	622
	Northwest	0.009	0.004	0.020	1496
	East	0.010	0.005	0.019	1666
	South	0.014	0.006	0.034	230
	Southwest	0.014	0.008	0.027	888
	Northeast	0.020	0.008	0.052	115
**Income**	Low	0.015	0.009	0.025	2409
	Medium	0.015	0.009	0.025	1458
	Upper medium	0.010	0.005	0.019	687
	High	0.006	0.002	0.014	333
**Family size**	Small	0.010	0.005	0.017	1557
	Medium	0.009	0.005	0.015	2306
	Large	0.014	0.008	0.024	1855
**Intended career**	Animal-related	0.009	0.004	0.020	378
	Medical/research	0.011	0.006	0.020	1572
	Education	0.010	0.005	0.018	418
	Other	0.013	0.007	0.022	2473
**Owned animals before 18 years old**	No ^a^	0.015	0.009	0.026	3335
	Yes ^b^	0.007	0.004	0.013	2460
**Currently owning animals**	No	0.010	0.006	0.017	4531
	Yes	0.011	0.006	0.020	1264
**Attended animal welfare lectures**	No ^a^	0.016	0.010	0.026	5192
	Yes ^b^	0.007	0.003	0.014	603
**Did laboratory work on animals**	No ^a^	0.014	0.008	0.025	4587
	Yes ^b^	0.008	0.004	0.014	1208

## Data Availability

The data presented in this study are available upon request from the corresponding author. The data are not publicly available due to privacy and ethical restrictions.
